# Factors Influencing the Implementation of Intimate Partner Violence Screening by Perinatal Obstetric Healthcare Providers in China: A Social–Ecological Perspective

**DOI:** 10.3390/healthcare14152286

**Published:** 2026-07-27

**Authors:** Mengyun Hu, Shuai Li, Jijie Chen, Wei Wang, Jiyun Wu, Yufeng Zhou, Yang Li, Xuekun Zhang

**Affiliations:** 1 School of Nursing, Suzhou Medical College, Soochow University, Suzhou 215006, China; 20234231010@stu.suda.edu.cn (M.H.); 20235231004@stu.suda.edu.cn (S.L.);; 2 The First Affiliated Hospital of Soochow University, Suzhou 215005, China; 3 School of Engineering, University of Liverpool, Liverpool L69 7ZX, UK; 4 Clinical Research Facility, Alder Hey NHS Children’s Foundation Trust, Liverpool L12 2AP, UK; 5 School of Nursing, the University of Texas at Austin, Austin, TX 78712, USA; yang.li@nursing.utexas.edu

**Keywords:** social–ecological model, intimate partner violence, obstetrics, qualitative research

## Abstract

**Introduction:** This study explores obstetric providers’ perceptions of intimate partner violence (IPV) screening in the Chinese clinical setting, guided by the social–ecological model. **Methods**: Purposive sampling was employed to recruit eight obstetricians and 12 obstetric nurses from a tertiary general hospital in Suzhou, Jiangsu Province, China. Individual semi-structured interviews were conducted between December 2024 and September 2025. **Results:** Guided by the social–ecological model, this study examines factors influencing healthcare professionals’ engagement in intimate partner violence screening across four levels. A multilevel framework was distilled; four key themes were identified, including the intrapersonal level, interpersonal level, institutional level and community and policy level. (1) The intrapersonal level—healthcare professionals’ competence (knowledge base, communication skills, role identity, screening attitudes, and diagnostic ability); (2) the interpersonal level—interdisciplinary collaboration (internal referrals and external support); (3) the institutional level—hospital screening efficacy (screening resources and management systems); and (4) the community and policy level—social environment (policy support, educational resources, and cultural context). **Conclusions:** Multilevel factors impact IPV screening in China. These results indicate that a multidimensional model of intervention should be designed to promote IPV screening in China, including integrating IPV curricula into mandatory medical student training, enhancing interdisciplinary collaboration, conducting public education on IPV, implementing socio-cultural adjustments, challenging authoritative attitudes, and providing comprehensive social support.

## 1. Introduction

Intimate partner violence (IPV) is one of the serious public health issues that not only severely impairs victims’ physical and mental health and quality of life but also triggers a range of adverse effects and poses substantial social risks [1]. IPV refers to violent behaviors occurring between intimate partners, between former partners, or within dating relationships, encompassing various forms such as physical, sexual, psychological, and economic abuse [2]. Epidemiological studies indicate a high prevalence of IPV worldwide. According to the National Intimate Partner and Sexual Violence Survey (NISVS) conducted by the Centers for Disease Control and Prevention (CDC) in the United States, approximately 41% of women and 26% of men have experienced such violence [3]. Studies in China also show that the probabilities of experiencing physical, psychological, and sexual violence in the general population range from 2.5% to 5.5%, 17.4% to 24.5%, and 0.3% to 1.7%, respectively, with lifetime prevalence rates warranting serious attention [4,5]. Although a recent study suggests a declining trend in the global incidence of IPV, this improvement is not uniform, and preventing and controlling IPV remain substantial challenges [1].

Intimate partner violence (IPV) exerts profound and complex impacts on victims’ health. Physical and sexual violence may directly result in physical injuries. Psychological and emotional abuse, including coercive control, verbal aggression and economic exploitation, can induce depression, anxiety, post-traumatic stress disorder and other mental problems, and even markedly raise victims’ suicide risk [6]. Different types of violent behaviors often co-occur and present obvious interactive effects.

Pregnancy increases vulnerability to IPV [7], and the impact during this period extends beyond the mother to affect the developing fetus. On the maternal side, IPV exposure is associated with increased mental health disorders and reduced engagement with prenatal and postnatal care. With respect to the fetus and child, substantial evidence links prenatal IPV exposure to low birth weight, preterm birth, shortened duration of breastfeeding, impaired mother–infant bonding, and early childhood behavioral and emotional difficulties [8,9]. In recent years, an association has also been identified between maternal IPV exposure and adverse neurophysiological outcomes in offspring, including altered auditory threat processing [10]. Collectively, these findings suggest that the effects of IPV during pregnancy may span across generations. Thus, the perinatal period represents a critical window for screening and intervention.

The World Health Organization recommends antenatal clinics as key venues for IPV screening and has established corresponding guidelines [11]. However, in clinical practice, particularly in low- and middle-income countries, the standardized implementation of IPV screening remains inadequate, highlighting a need for further research and practical exploration in this area [12]. In China, IPV screening has not yet been systematically implemented as a standardized routine within the national antenatal care system, and overall screening coverage remains low [4]. Furthermore, although China enacted the *Anti-Domestic Violence Law* as early as 2016, its specific implementation measures in healthcare settings have not been clearly defined, resulting in a significant gap between the legal framework and clinical practice [13]. Notably, the National Health Commission’s *Guidelines on Promoting the Development of Fertility-Friendly Hospitals* explicitly incorporate psychological and social factor assessments into maternal care services. This provides an important policy basis and a practical opportunity for integrating IPV screening into existing evaluation systems [5].

Over the years, a number of assessment tools have been developed in accordance with the diagnostic criteria of the *11th Revision of the International Classification of Diseases* (ICD-11) and the *Fifth Edition of the Diagnostic and Statistical Manual of Mental Disorders* (DSM-5), including the HITS (Hurt, Insult, Threaten, Scream) scale and the Woman Abuse Screening Tool (WAST). Designed to accommodate time constraints in routine clinical practice, these tools can comprehensively identify all forms of intimate partner violence (IPV), such as physical control, sexual control, psychological control and coercive control. Previous studies have indicated that these screening tools achieve sensitivity and specificity above 0.90 when applied to general populations [14]; therefore, they are applicable to the screening and detection of IPV in clinical settings.

Compared with international research, studies in China on IPV screening conducted by healthcare professionals remain relatively limited. Existing research predominantly focuses on quantitative surveys of knowledge and attitudes, while qualitative investigations exploring the facilitators and barriers influencing screening behaviors from a social–ecological perspective are notably lacking. Qualitative research, which aims to uncover subjective experiences and meaning-making through the analysis of participant narratives in natural settings, provides a critical pathway for revealing the underlying causes of complex clinical phenomena. This approach is particularly well suited to exploring the deeper determinants behind such multifaceted clinical issues.

A review of healthcare providers’ responses to IPV across global settings indicates that healthcare professionals—particularly those in obstetric care responsible for comprehensive health management during pregnancy—play an irreplaceable role in identifying, supporting, and referring IPV victims, making them well positioned to implement routine screening [15]. In practice, however, screening behavior is shaped by a complex interplay of factors operating across multiple levels. To examine this systematically, the present study draws on the social–ecological model proposed by [16] as its analytical framework.

This model proposes that health-promoting behaviors are shaped by the interaction of multiple factors. It was initially widely applied to guide the research design of health promotion programs. The original theoretical framework comprises five analytical levels. Combined with the practical context of clinical IPV screening, this study focuses on four core dimensions for systematic analysis, including intrapersonal factors (e.g., knowledge, attitudes, and self-efficacy), interpersonal factors (e.g., peer relationships and social support), institutional factors (e.g., organizational systems, resource allocation, and workplace norms), and community and policy factors (e.g., dominant social norms and regulatory systems). This theoretical framework enables the systematic analysis of key factors influencing clinical IPV screening and provides a solid theoretical foundation for the formulation of multidimensional targeted interventions and the optimization of IPV screening practices.

Therefore, this study adopts a qualitative research approach, guided by the social-ecological model, and employs semi-structured interviews to explore the perceptions and views of obstetric healthcare professionals in mainland China regarding IPV screening during pregnancy, as well as the influence of their environmental systems on screening practices. By examining the key factors affecting screening behaviors, this study aims to provide an empirical foundation for developing a systematic, culturally appropriate IPV screening and intervention strategy in China. The ultimate goals are to enhance the physical and mental health and quality of life of pregnant women while also contributing to policy development and promoting evidence-based clinical practice.

## 2. Methods

### 2.1. Study Design

This study adopts the descriptive qualitative research design proposed by [17]. Without preset research hypotheses, this method allows for an in-depth exploration of participants’ authentic perspectives and subjective experiences. The factors influencing obstetric clinicians’ implementation of IPV screening are multi-layered and context-specific, which cannot be fully and accurately captured by conventional quantitative questionnaire surveys. By contrast, descriptive qualitative research features flexibility and openness, closely aligns with real clinical scenarios, and enables the identification of implicit influencing factors. Such strengths effectively compensate for the limitations of quantitative research, making this design highly suitable for the present study. All findings are reported in accordance with the Consolidated Criteria for Reporting Qualitative Research (COREQ) to ensure the standardization and rigor of this qualitative study [18].

### 2.2. Study Participants

Participants were recruited through purposive sampling from a tertiary general hospital in Suzhou, Jiangsu Province. The inclusion criteria comprised the following:

(1) Holding a valid professional qualification certificate and having completed registration;
(2) Having worked in a clinical position within obstetric care settings (including obstetric wards, delivery rooms, or obstetric outpatient clinics) for one year or more;
(3) Willingness to participate voluntarily and to provide informed consent.

In China, resident physicians complete their medical license registration and hold formal clinical appointments. They are considered qualified clinical staff and are therefore eligible for inclusion.

The exclusion criteria were:

(1) Physicians and nursing staff in training status, defined as medical interns, nursing students, or those enrolled in standardized residency training programs.
(2) Staff who are not on duty for various reasons, such as maternity leave or sick leave.

The sample size was determined by data saturation, which was checked iteratively by two independent coders during the analysis. By the time we completed the 18th interview, no new themes, categories, or meaningful variations emerged across the four analytical dimensions. To confirm that saturation had been reached, we conducted two additional interviews. The final sample of 20 participants falls within the typical range for qualitative studies examining IPV-related cognitive domains, which is usually between 15 and 30 participants. Beyond saturation, we further assessed the sample using the information power framework proposed by [19]. This framework argues that sample adequacy depends not only on the number of participants but also on the interplay of five key elements: study aim, sample specificity, established theory, interview quality, and analytical strategy. Applying this framework to the present study: First, the study aim is clearly defined as exploring obstetric healthcare professionals’ perceptions and experiences of IPV screening within a specific institutional setting. The research questions are confined to specific research sites, participants and themes, which conform to the criterion that research objectives should maintain sufficient focus [20]. Second, Malterud et al. argued that participants’ characteristics are closely linked to research questions. All participants in the present study were recruited from the same tertiary hospital and engaged in maternal and child health care. Given the high specificity and homogeneity of the sample, the study achieved strong informational power. Third, this study adopted the social–ecological model, which has been widely utilized in qualitative research on perinatal health [21,22]. Malterud et al. explicitly pointed out that adopting established theories as the analytical framework for data analysis can strengthen the informational power of the sample. Fourth, participants in this study provided detailed and comprehensive descriptions of factors hindering IPV screening, which helps improve the informational validity of the sample. Fifth, this study strictly adopted the deductive qualitative analysis approach and analyzed the collected data via a top-down procedure. All coding results were reviewed and verified by members of the research team, with sound methodology and rigorous implementation throughout the research. Collectively, these measures strongly support the argument for sample adequacy. This study adopted maximum variation sampling, a form of purposive sampling, based on a pre-established three-dimensional sampling matrix covering clinical working experience, professional roles, and working settings. By maximizing sample heterogeneity, this approach enabled a comprehensive exploration of obstetric clinicians’ real experiences and multifaceted factors affecting IPV screening practices. A total of 20 female participants were enrolled in this study and were anonymously coded from N1 to N20. The participants were recruited from three clinical settings, including delivery rooms, obstetric wards, and outpatient departments, and covered three professional roles: obstetric physicians, obstetric nurses, and midwives. Their clinical working experience ranged from 1 year to more than 30 years, achieving sufficient variation across all sampling dimensions. Notably, the three participants working in delivery rooms were all midwives belonging to the nursing workforce. The demographic and professional characteristics of all participants are presented in Table 1.

### 2.3. Development of the Interview Guide

The research team conducted an in-depth analysis of the current prevalence of intimate relationship violence both at home and abroad through systematic literature retrieval. Following multiple discussions across the research team, the interview questions were systematically developed to ensure their meaningfulness, logical coherence, and clinical applicability, leading to the initial construction of the interview outline. Subsequently, two nurses from the obstetric ward were selected for pilot interviews. Based on the problems identified during the interviews, the outline was modified accordingly, and finally, a formal interview outline was formed. The main topic of the interviews revolved around “the cognitive attitude, practical ability and system implementation requirements of obstetric medical staff towards the screening behavior of intimate relationship violence among pregnant women”, specifically including: (1) training foundation and ability preparation; (2) understanding of the necessity and feasibility of screening; (3) obstacles and supporting conditions for clinical implementation; (4) response procedures for positive cases; and (5) supplementary opinions on IPV screening. For specific items, please refer to Attachment 1.

### 2.4. Data Collection

The research team consisted of one principal researcher and two postgraduate students in nursing. The principal researcher was an associate professor with a doctoral degree in nursing who specializes in women’s health, providing overall research design and academic guidance for this study. The two postgraduate students served as interviewers and co-researchers, both of whom received systematic professional training in nursing. Prior to data collection, the two students completed preliminary participant recruitment and follow-up liaison. All interviewers had no personal or professional interests related to the participants, which effectively reduced potential interview bias. Both interviewers received standardized specialized training covering core skills of semi-structured interviews, the definition of intimate partner violence, standardized IPV screening protocols, and the application of screening tools, ensuring the professionalism and standardization of the interview process. The two interviewers possessed complementary professional backgrounds. One student had completed clinical rotations at the study hospital and possessed solid obstetric clinical experience, enabling accurate identification of nonverbal cues and contextual implicit information during interviews, as well as facilitating trust establishment and positive interactions with participants. The other student, who was proficient in qualitative research methodologies without clinical practice experience, conducted interviews from an objective and independent stance. Through reflective questioning and neutral guidance, this interviewer offset the potential subjective predisposition caused by the clinical background of the first interviewer, thereby improving the objectivity and authenticity of the interview data.

This study consistently adhered to the reflexivity principle of qualitative research throughout the entire research process. Through researcher self-reflection and team-based quality control, subjective bias was minimized to ensure the authenticity and reliability of the findings. The primary interviewer had a clinical background in obstetrics and was familiar with clinical practices and real-world scenarios of IPV screening; therefore, the interviewer was able to capture nuanced details during the interviews. At the same time, given that the interviewer was not an active practitioner in the positions being studied, potential cognitive limitations arising from role-specific mindsets were effectively avoided. Nevertheless, the researcher’s clinical background may have induced social desirability bias among participants, leading some to deliberately conceal negative work experiences. To address this, neutral and non-judgmental questioning techniques were employed throughout the interviews, and discrepancies between participants’ subjective accounts and actual clinical practice were actively identified in order to reconstruct a more accurate picture of real working conditions.

All interviews were conducted in private and independent enclosed spaces within the hospital, with no third-party personnel present throughout the process, to maximize environmental privacy and ensure authentic participant responses. Prior to each interview, the researchers elaborated on the study procedures, research objectives, data usage and ethical requirements, addressed participants’ questions in a timely manner, and guaranteed that all original data, including interview recordings and transcribed texts, would only be used for academic research by the research team. Verbal informed consent was obtained from all participants before data collection. One-on-one interviews were performed based on a semi-structured interview guide, with each interview lasting 20–30 min and audio-recorded in full for raw data preservation. In addition, the interviewers observed and documented participants’ nonverbal information in real time, including tone of voice, facial expressions and body language, so as to improve the integrity and credibility of the research data.

### 2.5. Data Analysis

Directed content analysis [23,24] was selected as the analytical method, as the social–ecological model offers a theoretically sound and empirically supported framework for understanding the multilevel influences on IPV screening, making a deductive starting point both justifiable and appropriate. Interview recordings were transcribed verbatim within 24 h following each interview.

The analytical process comprised the following steps:

(1) Total immersion and familiarization with the data: The two coders thoroughly read through the transcripts to gain an overall understanding of the text content.
(2) Theory-guided deductive coding: This study employed the four-level framework of the social–ecological model [16], covering individual, interpersonal, organizational, community and policy domains, as the primary analytical structure. The researchers coded participants’ interview narratives into corresponding dimensions; for content beyond the predefined categories, new subcategories were established under the original hierarchical levels. To ensure the rigor and trustworthiness of the qualitative coding process, the following steps were undertaken. Two researchers independently performed calibration coding on a randomly selected subset of the interview transcripts [25]. Following this, they proceeded to code the entire textual dataset separately. Any coding discrepancies were addressed through a collaborative review of the original transcripts and iterative discussion until full consensus was achieved. On this basis, the entire research team subsequently conducted a collective audit of all codes to identify any missing codes and provide independent adjudications for controversial coding entries.
(3) Category refinement and subcategory development: The researchers grouped interview contents with similar meanings within each theoretical level and refined categories stepwise [23]. Correlations among subcategories were analyzed, and overlapping or ambiguous codes were reclassified to ensure the accuracy of all subcategories.
(4) Theme synthesis: The refined subcategories derived from the four levels were integrated into the social–ecological model for analysis and cross-checked against the original transcripts to ensure participants’ viewpoints were accurately captured [26].

In addition, to ensure reliability during the coding process, this study conducted an interrater reliability test [27]. The random number table method was adopted to select 4 interviewees (coded N2, N8, N12, N20) from all interview transcripts, accounting for approximately 20% of the total sample. The unit of analysis was defined as theme-related declarative sentences extracted from interview texts. Based on an established thematic framework, the first author and an independent third-party researcher not involved in the prior research independently coded the analyses separately, followed by a comparison of the consistency of thematic categorization between the two researchers. The calculation results showed that the coding consistency coefficient reached 0.719.

### 2.6. Selection of Participant Quotations

This study selects interview excerpts following three principles: typicality, richness and clarity. Typicality refers to screening statements that reflect the consensus of respondents from numerous interview viewpoints. Richness requires the selected excerpts to contain sufficient details and genuine emotions. Clarity means the chosen content is semantically explicit and easy to interpret [17]. In addition, this study also takes diversity and representativeness into account, namely, selecting statements that are limited in number yet distinctive and highly representative.

### 2.7. Translation Procedures

All interviews were conducted in Chinese. To ensure the quality of the manuscript, the research team first translated the finalized Chinese manuscript into English, after which a bilingual researcher with a professional background in nursing reviewed the translation. Team members then engaged in multiple rounds of cross-checking and reached a consensus on the wording. Throughout this process, particular emphasis was placed on preserving the original context and cultural nuances, ensuring that the translation was both semantically accurate and linguistically fluent.

### 2.8. Ethical Considerations

The study protocol was approved by the Ethics Committee of Soochow University (Approval No. SUDA20220609H01). All participant information was anonymized using numerical codes to protect confidentiality.

## 3. Results

We conducted top-down coding based on the four dimensions of the social–ecological model. The specific coding results are shown in Table 2.

### 3.1. Intrapersonal Level

#### 3.1.1. Inadequate Knowledge Reserve

The participants in this study consistently reported a lack of systematic training on intimate partner violence (IPV) in hospital settings. Furthermore, they indicated limited exposure to IPV-related awareness materials—including textual, visual, or video-based campaigns disseminated by institutions, communities, and broader society. As participant N3 noted, *“How can we ask questions without touching on sensitive or painful areas for pregnant women? How can we approach this topic in a way that feels comfortable for both the healthcare provider and the patient? I have no clear guidance on this.”*

#### 3.1.2. Low Self-Efficacy

Participants described that their limited knowledge and practical skills made them feel less motivated and less confident to conduct screening. As noted by N8: *“Our group of health workers may not be familiar with the screening techniques or solutions for this issue, let alone whether we can actually help resolve the pregnant women’s dilemmas. The question is how far you can go and how much you can actually help. If even the police often struggle to handle such cases, how can we manage?”*

#### 3.1.3. The Attitude–Practice Gap

Our study identified a marked disconnect between healthcare providers’ recognition of the importance of IPV screening and their actual screening practices. While participants widely acknowledged the necessity of screening—particularly in light of the established link between IPV and an increased risk of postpartum depression—the majority reported a tendency to avoid screening in practice, citing insufficient screening or response to positive cases. As expressed by N17: *“Screening is important because IPV can greatly raise the risk of postpartum depression. Yet, it often feels impractical. Even if we detect a positive case, what can we realistically do to help? I can’t act like a clown performing tricks—doing my core job well is already a challenge.”*

#### 3.1.4. Perceived Non-Duty of IPV Screening in Healthcare Practice

A subset of senior healthcare professionals, particularly physicians, displayed marked role ambiguity and a tendency to deflect responsibility in relation to IPV screening. They frequently regarded IPV as a distinct “non-medical issue” and did not consider screening to be part of their routine clinical duties, resulting in deliberate avoidance of this task. As articulated by participant N18: *“Hospitals are meant for treating illness and saving lives. Even if a pregnant woman screens positive during her consultation, I can only address her physiological needs. Screening for IPV is not the doctor’s role—it is more suitably handled by nursing staff.”*

#### 3.1.5. Subjective Doubts Toward IPV Screening Authenticity and Efficacy

During the interviews, healthcare professionals voiced measured skepticism about the authenticity of IPV screening and the likelihood of detecting positive cases. The establishment of trust between the screener and the screened individual is considered a key barrier to the truthful disclosure of information. Interviewees emphasized that building reliable trust between healthcare providers and pregnant women is a fundamental prerequisite for encouraging self-disclosure. In perinatal outpatient settings, however, brief consultation times, high patient turnover, inconsistent staffing, and generally weak interpersonal connections make it difficult to develop substantial trust. N16 noted: *“How can we be sure that the answers a pregnant woman gives are truthful? If she does not have even basic trust in you, why would she tell you the truth?”*

Furthermore, individualized perceptions of privacy protection significantly inhibit pregnant women’s willingness to disclose intimate partner violence (IPV). Participants widely regarded IPV as a deeply personal matter and noted that concerns about family reputation and social judgment often lead women to deny such experiences. As N2 articulated, *“This is a private family matter. It is unlikely that a pregnant woman would disclose the truth. Even I, if in her position, would hesitate to speak openly about it.”*

Lastly, skepticism regarding screening efficacy prevailed among some healthcare providers. This perspective is illustrated by N17′s comment: *“How many positive cases can we realistically expect to identify? The very fact that these women continue to attend prenatal visits and plan for childbirth suggests their marital relationship is not severely strained. If serious IPV were present, would they even seek regular medical care?”*

#### 3.1.6. Objective Limits Toward IPV Screening Effectiveness and Accuracy

Interviewees pointed out that a single, static screening may be inadequate for accurately distinguishing between long-standing patterns of violence and occasional conflicts, thus failing to capture the dynamic nature of abusive relationships. As noted by N18: *“I think relying on just one inquiry during a prenatal visit is not very reliable. It’s possible that when you ask her, she might be particularly upset and describe ordinary disagreements as very serious. In such a case, can a positive screen really be considered valid?”* Participants raised concerns that heightened emotional sensitivity during pregnancy might lead women to describe ordinary conflicts as violence. N20 remarked: *“During pregnancy, hormone levels are very unstable, and emotions are naturally more sensitive. A couple might just have a minor argument—something that would normally be insignificant—but at this time she may feel deeply wronged and perceive it as violence.”*

#### 3.1.7. Perception of Safety Risks as a Barrier

Participants indicated that concerns for personal safety were also a reason for avoiding or limiting follow-up activities related to IPV. As N9 stated: “*Last time we followed up on a case, the male partner yelled and shouted at us. For several days afterward, I was afraid to come to work, constantly worried that he might retaliate against me*.” N17 added: *“But what if we are threatened during follow-up calls or community visits? I don’t want to invite trouble.”*

### 3.2. Interpersonal Level—Gaps in Interdepartmental Support Systems

#### 3.2.1. Lack of Within-Hospital Interprofessional Collaboration

Participants described a lack of cross-departmental support as a barrier to IPV screening follow-up. Several providers expressed uncertainty about what to do after identifying a positive case and reported feeling unsupported in the absence of clear referral pathways. As participant N8 explained: *“Our primary concern is the lack of follow-up* *support for women who screen positive. We are afraid that after identifying a case, we won’t know what to do next. Without a strong institutional support system, we feel profoundly isolated, and the sustainability of screening is significantly undermined.”*

#### 3.2.2. Disruption of Extramural Integrated Networks

Participants described limited communication and coordination between their clinical settings and external support systems, including the police and community services. Several providers noted that after identifying a potential IPV case, there was no clear pathway for referral or information sharing with these external agencies. As N8 described, *“The biggest problem is the ‘disconnection.’ The obstetrics department reports a case, the police come, but then what? After the patient returns to the community, is there any follow-up? Has the perpetrator been effectively warned? We have no idea. The police don’t provide us with feedback, and the community doesn’t initiate contact with us either.”*

### 3.3. Institutional Level—Hospital Level Factors

#### 3.3.1. Insufficient Allocation of Screening Resources (Including Space, Personnel, and Time)

Participants identified three major resource-related barriers to IPV screening: the lack of private screening spaces, insufficient screening personnel, and time constraints.

Multiple participants described the obstetric outpatient setting as a barrier to screening. Clinics were reported to be generally open, with family members and others frequently accompanying pregnant women into consultation rooms. Participants noted that no dedicated private screening space had been designated, nor had encrypted access controls been implemented to protect personal information. As N7 stated: *“The hospital and department have not provided a dedicated screening space for pregnant women. Especially when her husband is present, conducting screening in such an open setting may lead the pregnant woman to refuse screening out of fear of displeasing her partner.”*

Participants also identified staffing shortages as a barrier. It was reported that the number of healthcare professionals possessing both screening competence and willingness is limited. As N10 explained: *“Hospitals would need to regularly assign dedicated screening personnel to carry out this program without disrupting routine departmental workflows. However, given the current situation, the hospital lacks the human resources for such screening initiatives.”* N12 added: *“Ward duties are already demanding. Every staff member has their own tasks to complete. It is unrealistic to assign a dedicated clinician to sit in the ward and screen pregnant women.”*

Time constraints within routine clinical workflows were also reported as a barrier. Participants described compact outpatient schedules, limited time per consultation, and high daily patient volumes, which left little room for the extended conversations required for screening. As N5 noted: *“Healthcare workers are routinely too busy; beyond their core duties, there is simply no extra time to sit down and have such extensive conversations with potential victims.”* N8 added: *“The outpatient clinic sees a high volume of patients daily. The time we can allocate to each pregnant woman is limited. This issue cannot be resolved with just a couple of quick questions. To do it properly, you need to invest time and effort. But the reality is, if you spend that much time on one individual, what happens to the other pregnant women waiting?”*

#### 3.3.2. Deficiencies in Management Systems: Inadequate Prioritization and Security Safeguards

Participants reported that deficiencies in hospital management systems hindered IPV screening. First, participants described issues related to prioritization. Interviewees consistently reported that the degree of attention and support from hospital leadership affected the implementation and sustainability of IPV screening. According to participants, screening for intimate partner violence currently lacks formal institutional authorization, is not integrated into performance metrics, and is not linked to any material incentives. As N3 pointed out: *“All our tasks require approval from leadership before they can proceed. This screening is not mandated by the hospital, and currently there are no protocols specifying how we should conduct it within the screening process.”* As N18 noted: *“Currently, this screening is not part of our routine responsibilities. If it is considered additional work, will the managers provide us with an allowance or compensation? Without corresponding material incentives, it is unlikely that many would be willing to carry it out.”*

Second, participants raised concerns about inadequate security safeguards. Interviewees highlighted that a well-established institutional safety framework is important for healthcare providers conducting IPV screening. Within the current clinical context, participants regarded IPV screening as a sensitive undertaking with inherent risks. As articulated by N11: *“Does the hospital have a protective protocol for these situations? What should we do if a perpetrator confronts us during the screening process? Without the hospital providing a safety net, neither medical staff nor pregnant patients feel secure enough to engage with this sensitive topic.”*

### 3.4. Community and Policy Level—Social Factors

#### 3.4.1. The Constraining Influence of Cultural Norms

(1) **Traditional Views towards IPV.**

Participants were widely influenced by traditional social perceptions that categorize intimate partner violence (IPV) as a “household matter” or private conflict, rather than a systematic public health and social safety issue. As explained by N7: *“There is an old Chinese saying: ‘Even an honest official finds it hard to settle family disputes.’ IPV is considered an internal family affair; outside involvement is often seen as inappropriate.”* Participants noted that cultural norms discouraged the open discussion of family matters, leading some women to conceal their experiences. As N2 stated: *“Chinese cultural education has long emphasized that women should learn to endure and tolerate. As a result, most Chinese women tend to remain silent in such situations. Deeply influenced by traditional norms, they are unlikely to proactively disclose their experiences.”*

(2) **Legitimization of Violence in Contexts of Power Imbalance.**

Interviewees highlighted that within China’s traditional patriarchal social structure, enduring gender-based power disparities typically afford men greater decision-making authority and dominance in domestic settings, while women are often positioned in comparatively subordinate roles. As one participant (N5) remarked, *“In many Chinese families, the husband holds ultimate authority. Even when subjected to unjust treatment, pregnant women may feel unable or unentitled to resist. Over time, they may cease to perceive such situations through a rational lens. More troublingly, even family members and friends often rationalize intimate partner violence.”*

(3) **Stigmatization of Violence.**

In this study, healthcare professionals noted that beyond cultural restraints on disclosure, society also attaches negative labels to “domestic violence.” As N12 explained, *“The social stigma surrounding IPV—such as being labeled ‘incompetent’—is also a significant factor. Pregnant women may feel deeply ashamed to speak out.”* N13 added, *“Some even worry that revealing their abuse would lead to discrimination or ridicule from other pregnant women or from healthcare staff themselves.”*

#### 3.4.2. Deficiencies in Social Support Systems

(1) **The Constraints of Financial Dependence.**

A prevalent theme in the interviews was the fear among victims that resistance could lead to the termination or reduction in financial support from their husbands, effectively trapping them within the abusive relationship. As N7 articulated: *“The primary reason many pregnant women remain silent is fundamentally economic. Without independent income or employment, they are entirely financially reliant on their spouses. The prospect of confronting their abuser—and potentially facing retaliatory financial withdrawal—leaves them feeling they have no alternative but to endure the abuse.”*

(2) **Fear of Escalating Violence.**

Participants reported that pregnant women were often reluctant to disclose IPV due to concerns that seeking help would not lead to effective protection but might instead provoke further violence from their partners. The healthcare professionals interviewed reported that pregnant women actively assess these systemic gaps—evaluating whether existing safeguards (e.g., police intervention, medical confidentiality) can mitigate risks. For instance, N15 noted: *“Many pregnant women have expressed sentiments such as, ‘What purpose does reporting serve? Police interventions are often merely procedural. Once they depart, the violence I face may become more severe.’ Their reluctance stems not from unwillingness to seek help, but from the fear that action could provoke partners’ anger without ensuring effective protection.”*

#### 3.4.3. The Implementation Gap in Legal Systems

Participants described a gap between existing legal provisions and their practical enforcement as a barrier to IPV screening. Although China’s Anti-Domestic Violence Law establishes institutional mechanisms—including mandatory reporting, written warnings, and personal protection orders—several participants reported difficulties in applying these provisions in clinical practice. As N2 explained: *“While the Anti-Domestic Violence Law is widely recognized, its implementation remains fraught with difficulties. Physical injuries on pregnant women may heal before they can be formally documented by medical professionals, and psychological violence is even harder to substantiate.”*

### 3.5. Barriers to Violence Recognition

#### The Tendency Toward Internal Attribution in Victims of IPV

Participants observed that some pregnant women tended to blame themselves for the violence they experienced. Rather than attributing responsibility to the perpetrator, they engage in intense self-scrutiny, interpreting their partner’s violent behavior as a result of their own perceived shortcomings, thereby engaging in self-reproach. As N8 expressed, *“What is most heartbreaking is that these pregnant women, after being physically abused, react not with anger but with self-blaming. They wonder, ‘Was I too irritable during pregnancy?’ or ‘Did I fail to take care of the family properly?’ You see, fundamentally, it’s because our publicity and education are inadequate—we haven’t given them support, so they turn blame inward.”*

## 4. Discussion

### 4.1. Intrapersonal Level

#### 4.1.1. The Dual Burden of Awareness Without Competence

This study reveals a pronounced structural disconnection between knowledge and skills in IPV screening among perinatal healthcare professionals. The systematic lack of training has resulted in an ambiguous understanding of screening methods and interventions, thereby undermining both recognition ability and self-efficacy in clinical practice [28]. Participants linked their limited IPV-related knowledge and practical skills to a reduced sense of motivation and confidence in conducting screening. This finding aligns with previous research indicating that insufficient IPV-related knowledge and training among healthcare workers constitute a key barrier to effective screening and assistance [4]. Despite the WHO’s emphasis on the necessity of adequate training for healthcare providers, relevant education remains generally inadequate or limited on a global scale [29].

Such training gaps directly contribute to a “know–do gap”: although most healthcare professionals acknowledge the importance of IPV screening in preventing adverse outcomes such as postpartum depression, insufficient perceived intervention competence significantly hampers the implementation of screening in clinical settings. This echoes the conclusion drawn by Derksen et al. that a lack of intervention capacity restricts the integration of screening into routine practice [30]. Bandura’s self-efficacy theory provides a compelling framework for explaining the knowledge–practice gap observed among healthcare providers in IPV screening. When practitioners lack both the professional competence to identify intimate partner violence and the confidence to navigate subsequent intervention procedures and case management, the negative outcomes they perceive—such as increased workload, limited consultation time, and a sense of helplessness stemming from the belief that “screening without adequate follow-up support cannot truly help patients”—tend to outweigh their anticipated benefits of screening, thereby leading them to avoid engaging in this practice.

The findings of this study indicate that participants demonstrated notable deficiencies in both knowledge and skills, highlighting an urgent need for IPV-related training in this region. Enhancing the effectiveness of IPV screening requires not only the updating of existing knowledge but also the implementation of systematic skills training and the cultivation of a supportive practice environment [11]. Future research may further explore the feasibility of scaling such initiatives nationwide and establishing a systematic pathway for capacity building—for example, by integrating scenario-based simulations and case discussions into structured curricula across both medical education and in-service training. Core competencies such as risk assessment, communication, safe referral, and psychological support must be strengthened. These measures will solidify healthcare professionals’ competency and confidence in conducting IPV screening, thereby bridging the gap between awareness and practice [5,31].

#### 4.1.2. The Entrenchment of Professional Roles

This study further reveals that some clinical practitioners, particularly physicians, exhibit a tendency to narrowly define their roles and solidify responsibility boundaries regarding IPV screening. They often perceive IPV screening as a “social issue” or within the nursing domain, rather than as part of their professional duties. This phenomenon is not uncommon in clinical practice. For example, one study indicates that nearly half of primary care physicians do not explicitly include IPV screening within their defined professional responsibilities [32]. This reflects how, in a disease-centered clinical environment, IPV screening—due to its perceived “non-urgent” and “non-pharmacological” nature—is often marginalized and subsequently categorized as nursing or humanitarian care [12].

To shift this cognitive and practical status quo, future efforts should promote structured improvements through system-level restructuring and individual empowerment. On the one hand, interprofessional collaboration mechanisms should be established to clarify the roles of physicians, nurses, and other health professionals in screening, assessment, referral, and support [33], thereby expanding healthcare providers’ understanding of their own responsibilities. On the other hand, IPV screening and intervention should be integrated into the fundamental framework of healthcare delivery at the systemic level. This would guide healthcare professionals—especially physicians—to internalize screening as a professional responsibility that spans the entire continuum of care, thereby encouraging their role transition from mere disease treaters to advocates and collaborators who holistically address patient safety, health, and social well-being.

#### 4.1.3. Trust, Privacy, and Efficacy: Unpacking the Rational Hesitancy in IPV Screening Practice

The findings indicate that healthcare professionals exercise a form of prudential pragmatism regarding intimate partner violence (IPV) screening. Their screening practices are embedded within and shaped by an interrelated triad of factors: the therapeutic alliance (trust), contextual privacy concerns, and perceived professional efficacy.

In particular, current obstetric practice is often characterized by superficial clinician–patient interactions and fragmented care delivery, which collectively undermine the establishment of effective trust. Evidence indicates that sustained attentiveness, empathy, and a non-judgmental attitude among healthcare providers are central to building therapeutic trust [34]. Therefore, adopting a continuity-of-care model based on consistent clinical teams—such as obstetric case management and scheduled follow-up—can help patients develop emotional attachment and a sense of safety, thereby creating a crucial foundation for subsequent IPV screening. This finding aligns with the conclusions drawn by Corrine Lu et al. [5]. Accordingly, it is recommended that healthcare institutions systematically implement a continuity-of-care model to gradually develop professional, caring, and trusting relationships with individuals affected by violence, ultimately enhancing the authenticity and effectiveness of IPV screening.

This study also identified protective perceptions of privacy among healthcare providers as a significant barrier to IPV screening. Clinicians often regard IPV as a “private family matter” and assume that pregnant women may deny experiences of violence to protect their reputation. This reflects the tension between their dual roles as professional interveners and social members, creating an ethical and practical conflict between safeguarding patient privacy and conducting necessary clinical inquiry. Similarly, a study of U.S. primary care physicians noted that concerns about patient privacy are a key reason for not routinely implementing IPV screening [35].

These findings suggest that the privatization of IPV is a widespread perception that substantially impedes screening in clinical practice [5,28,32]. Therefore, future interventions should systematically integrate trauma-informed care principles into IPV-related training to enhance the professional legitimacy of screening. Through standardized communication skills training, healthcare providers can reframe their understanding of IPV screening—from viewing it as “intrusion into privacy” to recognizing it as “a routine professional health risk assessment.” [31,33].

This study further reveals a critical misconception in clinical practice: the tendency among some healthcare providers to equate regular antenatal care attendance with the absence of intimate partner violence. This flawed assumption arises from two interrelated issues. First, it reflects a narrowly utilitarian understanding of screening value, where effectiveness is judged primarily by detection rates, thereby overlooking the broader clinical role of screening in health education, safety awareness, and trust building. Second, it oversimplifies the lived experience of pregnant women who are subjected to violence, where the tendency among some providers is to interpret regular antenatal attendance as evidence that a patient’s relationship is stable and free of violence. Underlying this assumption is what might be described as an “ideal victim” schema—an implicit expectation that women experiencing severe IPV would not, or could not, maintain consistent engagement with healthcare services. This reasoning does not hold up empirically. Research on help-seeking behavior among IPV survivors suggests that regular prenatal visits may represent one of the few safe and accessible points of contact available to them [36]. Clinicians who view compliance as safety are most likely to overlook the women most in need of being reached.

In addition to concerns about screening detection rates, respondents also questioned the reliability of screening results. A study indicated that existing screening tools generally have favorable specificity. However, their screening efficacy is easily affected by factors such as study population, cultural background and research setting, resulting in varying sensitivity and specificity [37].

Therefore, there is a clear need to reframe how the effectiveness of IPV screening is conceptualized and evaluated. The focus should shift from a detection-centered model to a holistic clinical practice that integrates education, risk assessment, and therapeutic engagement. It should be emphasized that screening serves only as the first step in a two-stage assessment process and is not a final diagnostic tool. Even when screening does not identify violence, a respectfully conducted assessment combined with supportive counseling represents a meaningful clinical intervention that fulfills important preventive and relational functions. Given that findings from a single screening cannot serve as a clinical diagnostic criterion, medical staff should conduct multiple repeated screenings for pregnant women throughout the entire perinatal period and postpartum phase. In-depth interviews should be supplemented to identify high-risk pregnant women and improve the reliability of screening results.

#### 4.1.4. Occupational Safety Concerns as a Profound Deterrent to Screening Motivation

This study also identified concerns about personal safety among healthcare providers as a significant barrier to implementing IPV screening and intervention. However, healthcare institutions often lack adequate risk anticipation and have not established corresponding systemic safeguards to support clinicians in this role. Bianco [38] likewise note that workplace violence represents a serious threat to clinicians’ well-being, potentially increasing occupational exposure risks and exacerbating feelings of insecurity during screening encounters.

Based on the above findings, the researchers suggest that future efforts of healthcare organizations may consider systematically developing integrated safety support systems. This should include the installation of intelligent alert devices and real-time monitoring in clinical areas, along with optimized emergency response protocols for high-risk situations. Furthermore, the formation of multidisciplinary rapid response teams—comprising security personnel, clinicians, and social workers—and the implementation of standardized safety plans would provide a solid institutional and physical foundation, enabling healthcare professionals to conduct IPV screening with greater confidence and protection.

### 4.2. Interpersonal Level

#### Structural Breakdown in Cross-Team Collaboration

Both the lack of sustained institutional support within hospitals and the discontinuity of external support networks emerged in this study as critical factors hindering healthcare professionals from effectively carrying out IPV screening. Najafi M. notes that establishing a multi-sectoral collaboration mechanism for IPV screening can significantly enhance the sustainability of such initiatives [39]. Correspondingly, a study conducted in Hong Kong emphasized that IPV is a complex issue intersecting health and social domains [40] and that screening based solely within the hospital system is insufficient for comprehensive identification and intervention. Therefore, improving IPV outcomes requires coordinated efforts between healthcare institutions and broader society beyond interdepartmental collaboration within obstetric settings.

Based on these findings, the researchers suggest that healthcare institutions may consider establishing intra-hospital, cross-departmental coordination mechanisms to ensure a clear referral pathway once violence is identified—where clinicians have “somewhere to refer and someone to rely on” [31,32]. Existing evidence links prenatal exposure to intimate partner violence (IPV) with adverse health outcomes in infants and young children. In light of our study’s finding that obstetric clinicians face barriers to routine IPV screening, we propose an exploratory recommendation to incorporate child health professionals into interdisciplinary IPV screening teams to facilitate case identification of IPV-affected families. Concurrently, hospitals may further consider collaborating with community organizations, women’s federations, public security, and judicial departments to develop integrated “one-stop crisis centers.” Such models can establish a seamless support chain that bridges medical detection, social intervention, and legal protection. Furthermore, pregnant women should be connected with accessible resources—such as temporary housing, legal aid, and psychological support—and actively involved in co-developing individualized safety plans. For those identified as experiencing violence, structured longitudinal follow-up is needed to continually assess the physical and mental health of both the mother and child, ensuring that interventions remain safe and effective over time.

### 4.3. Institutional Level

#### 4.3.1. Resource Scarcity as a Systemic Constraint on Screening Practice

This study identified several key barriers to IPV screening, including open screening environments, insufficient staffing and time constraints. Heavy workloads and limited time hinder healthcare professionals’ capacity to conduct IPV screening and manage related cases effectively [12].

This finding provides evidence that can support medical institutions in developing dedicated or interdisciplinary screening teams in future practice. Intimate partner violence (IPV) screening must be conducted through comprehensive measures, including ensuring a private screening environment, implementing policies to ensure patients are seen alone (without partners or family members present), and employing personal information encryption to safeguard confidentiality [36] Furthermore, the research team puts forward a suggestive proposal to integrate IPV screening with routine psychological assessments to optimize the screening workflow and improve overall efficiency. Future research may be conducted in perinatal clinical settings to compare various staffing configurations and workflow integration approaches and identify feasible implementation strategies for IPV screening.

#### 4.3.2. Systemic Barriers in Screening Practice

This study found that hospital management’s emphasis and institutional support are crucial for institutionalizing IPV screening, a finding consistent with existing research [41] However, clinical practice reveals that IPV screening heavily relies on the initiative of healthcare professionals and is often hindered by time constraints, staffing shortages, lack of incentives, and safety concerns. Therefore, we recommend that healthcare institutions adopt a dual strategy. First, integrate screening into routine protocols and performance evaluation systems through official policy, provide material incentives, and grant formal authorization. Second, uphold the principle of “equal emphasis on screening and protection” by deploying dedicated security teams and establishing emergency response plans to ensure the safety of both patients and staff [11]. This approach will systematically enhance engagement and ensure the sustainability of screening programs.

### 4.4. Community and Policy Level

#### 4.4.1. Cultural Adaptation and Reconstructing Misconceptions

This study reveals that implementing intimate partner violence (IPV) screening within China’s traditional socio-cultural context presents significant challenges. Deeply internalized beliefs, such as “family shame should not be publicized” and “the husband is paramount,” not only rationalize IPV but also substantially reduce victims’ acceptance of screening initiatives. Furthermore, the stigma associated with IPV intensifies the psychological pressure on victims to remain silent. The interplay between these cultural norms and social structures collectively shapes how victims perceive and respond to violence.

Notably, the cultural phenomena cited by respondents in this study, such as norms of female forbearance, a focus on family privacy and patriarchal authority, are not unique to Chinese society. This finding resonates with cross-cultural research. For instance, Dietrich and Schuett’s [42] work on “culture of honor” indicates that preserving family reputation may take precedence over personal safety. Similarly, Disanayake’s [43] scoping review systematically identifies patriarchal stigma centered on “honor” as a key barrier to help-seeking. In addition, this structural power imbalance, deeply embedded in socio-cultural norms, not only reinforces established patterns of interaction within households but also fundamentally undermines the equitable foundation for intimate relationships. This phenomenon can be interpreted through Bourdieu’s [44] theory of masculine domination. From the perspective of this theory, gender inequality stems not merely from individual perceptions but from a socially constructed power system. Within such a system, intimate partner violence functions as an implicit vehicle sustaining masculine domination and consequently becomes normalized.

The findings of this study suggest that IPV screening and intervention need to account for region-specific factors, which share cross-cultural commonalities yet display distinct local manifestations. This observation provides evidence for developing future IPV prevention strategies in China that adopt a culturally sensitive framework to advance public awareness while respecting contextual realities. Future efforts may further consider integrating IPV screening into routine prenatal care pathways to normalize screening and mitigate associated stigma. Second, there is a need to develop and validate culturally adapted screening tools. Incorporating an assessment of “concerns about family honor or social judgement” as a distinct dimension could be pivotal [45]. Finally, and crucially for nursing practice, healthcare professionals must receive training to enhance their cultural competence. They need to recognize the significant variations in understanding and acceptance of IPV screening among victims from different ethnic and regional backgrounds. Employing culturally congruent communication strategies during screening is essential to improving its acceptability, accuracy, and long-term sustainability.

#### 4.4.2. Limitations of Inadequate Social Protection

The findings of this study reveal that gaps in the social protection system constitute an insurmountable barrier for victims. Economic vulnerability places pregnant women in a position of dependency on their partners [36], while ineffective police interventions erode trust in social support mechanisms, with victims fearing that seeking help may exacerbate violence rather than resolve it. This phenomenon of systemic response failure is not unique; international research indicates that police may be reluctant to conduct thorough investigations in IPV cases due to high workloads, professional burnout, or beliefs in victim-blaming narratives [46].

The findings of this study suggest that China currently lacks comprehensive institutional norms and response systems for survivors of IPV, which may hinder victims from accessing effective protection. While this is a single-center study with a limited sampling scope, the results remain of great reference value and can serve as preliminary evidence for the development of future support systems. Specifically, a sound social security system could be established in the future, including creating a special support fund for pregnant women and enforcing gender equality policies in employment to alleviate economic dependency. Furthermore, systematic, victim-centered training for police and social workers is essential to shift attitudes and improve responses. Finally, institutionalized collaboration mechanisms must be strengthened to ensure seamless coordination and continuous follow-up among law enforcement, judicial bodies, and social services, thereby forming a sustained, closed-loop protection chain.

#### 4.4.3. The Erosion of Legal Efficacy

This study further reveals that inadequate evidence collection and ambiguous legal standards are critical barriers to effectively holding perpetrators accountable. These findings are consistent with the existing literature, which indicates that even in countries with well-developed anti-domestic violence legislation, insufficient evidence remains a primary reason for failed prosecutions or victim withdrawal [13]. This phenomenon is particularly pronounced in China, likely due to the relatively high evidentiary thresholds, cumbersome judicial procedures, and lack of detailed operational standards in legal assessments, all of which jointly hinder the judicial recognition of IPV.

Although this study employed a single-center design, which limits the generalizability of the findings, participants’ accounts of gaps in law enforcement suggest that lowering evidentiary standards and streamlining judicial procedures in IPV-related cases may represent important agenda items for future policy development and research. The findings may also have preliminary reference value for the future refinement of IPV-related laws and regulations. For instance, it is recommended that China should work toward lowering the evidentiary burden in IPV cases and simplifying related judicial procedures. Additionally, establishing efficient and standardized injury documentation protocols within healthcare settings is essential to provide reliable evidence for legal intervention, ensuring that protective measures can be effectively implemented.

#### 4.4.4. The Impact of Societal Education Gaps on IPV Recognition

This study indicates that the lack of societal education regarding intimate partner violence (IPV) is a significant factor contributing to the diminished ability of pregnant women to identify IPV and their tendency toward self-blame [47], which aligns with the findings reported by Wenham [40]. This finding highlights a notable gap in current health education systems: within a social environment where public discourse on IPV remains limited, victims often not only struggle to recognize abusive behaviors but are also more likely to blame themselves, thereby remaining trapped in high-risk relationships.

Therefore, future efforts could consider establishing a multilevel, comprehensive, society-wide IPV education framework. By leveraging social media, community-based campaigns, and targeted health communication strategies, this initiative should unequivocally convey the message that “no form of violence is acceptable.” Such efforts are essential to empower victims with the awareness and agency necessary to recognize and confront IPV, ultimately promoting early intervention and support-seeking behaviors.

## 5. Conclusions

This study, grounded in the social–ecological model, reveals that the implementation of IPV screening by obstetric healthcare providers is hindered by a complex interplay of factors across intrapersonal, interpersonal, institutional and community and policy levels. This multilevel interdependence necessitates integrated, systems-oriented interventions rather than isolated solutions.

## 6. Limitations

Several limitations of this study warrant consideration.

First, regarding study design, this study was conducted at a tertiary hospital in Suzhou, Jiangsu Province, without incorporating screening perspectives from medical staff across diverse socio-cultural backgrounds and geographic regions. Whether the present findings can be generalized to IPV screening practices in primary-level hospitals or settings with varied socioeconomic conditions remains to be further explored.

Secondly, this study is limited by sample homogeneity. All interviewees in this research are female healthcare professionals. Given that male medical staff may hold distinct perceptions and attitudes toward screening for intimate partner violence, the exclusive recruitment of female participants could lead to one-sided research conclusions.

Third, regarding methodological considerations, two researchers performed independent coding and inter-coder reliability assessment in this study. Our research team resolved coding discrepancies. However, member checking with participants, complete audit trail documentation and negative case analysis were not conducted, which may impair the trustworthiness of the qualitative data to a certain extent.

## 7. Implications for Practice and/or Policy

Future research should involve multicenter designs and include male healthcare providers from regions with varying socioeconomic profiles. Particular attention should be paid to how physicians’ perception of roles and screening attitudes directly influence screening effectiveness. Pilot programs that link screening with coordinated follow-up interventions should also be implemented to assess real-world effectiveness. Furthermore, in-depth, semi-structured interviews with pregnant women who screen positive for IPV are recommended to further elucidate the complex barriers to implementation, validate and refine the proposed framework, and enhance the external validity of the conclusions. Consistency checks, member checking, audit trails, and negative case analysis should also be integrated into the study to improve methodological rigor and strengthen the credibility of the findings.

## Figures and Tables

**Table 1:** Demographic and professional characteristics of obstetric medical staff.

Characteristic	Number	% (n)
**Gender**		
Female	20	100.00
**Age**		
≤30	3	15.00
31~40	9	45.00
41~50	6	30.00
≥51	2	10.00
**Educational Levels**		
Secondary	1	5.00
Junior college	1	5.00
Undergraduate	9	45.00
Master	9	45.00
**Professional Title**		
Nurse	4	20.00
Supervisor nurse	3	15.00
Associate chief senior nurse	3	15.00
Chief senior nurse	2	10.00
Chief physician	3	15.00
Resident doctor	4	20.00
Physician-in-charge	1	5.00
**Working Section**		
Delivery room	3	15.00
Maternity ward	10	50.00
Obstetric outpatient	7	35.00
**Years of working**		
≤10	8	40.00
11~20	7	35.00
≥21	5	25.00

**Table 2:** Obstetric providers’ views on screening for intimate partner violence and related factor dimensions.

Main Categories	Generic Categories	Initial Categories	Participants (n)
Intrapersonal Level	Individual Healthcare Provider Factors	Inadequate Knowledge Reserve	16
		Low Self-Efficacy	4
		The Attitude–Practice Gap	6
		Perceived Non-Duty of IPV Screening in Healthcare Practice	4
		Suspicion toward IPV Screening Process and Validity: (1) Subjective Doubts Toward IPV Screening Authenticity and Efficacy.	6
		(2) Objective Limits Toward IPV Screening Effectiveness and Accuracy.	5
		Perception of Safety Risks as a Barrier	3
Interpersonal Level	Gaps in Interdepartmental Support Systems Interprofessional and External Collaboration Gaps	Lack of Within-Hospital Interprofessional Collaboration	6
		Disruption of Extramural Integrated Networks	10
Institutional Level	Hospital Level Factors Organizational-Level Barriers	Insufficient Allocation of Screening Resources (Including Space, Personnel, and Time)	10
		Deficiencies in Management Systems	5
Community and Policy Level	Social Factors Socio-Cultural and Structural Factors	The Constraining Influence of Cultural Norms	9
		Deficiencies in Social Support Systems	6
		The Implementation Gap in Legal Systems	4
		Inadequate Societal Education	5

## Data Availability

The data that support the findings of this study are not publicly available due to ethical and privacy restrictions. This research is a qualitative study based on interview transcripts, which contain potentially identifiable personal information. Participants were informed that their data would be used solely for the purposes of this study and did not provide consent for public data sharing. Consequently, the dataset cannot be deposited in a public repository. Should the journal require further clarification or alternative data deposition arrangements, the first author is available to accommodate all editorial requirements and may be contacted for any additional information.
